# The Involvement of Intestinal Tryptophan Metabolism in Inflammatory Bowel Disease Identified by a Meta-Analysis of the Transcriptome and a Systematic Review of the Metabolome

**DOI:** 10.3390/nu15132886

**Published:** 2023-06-26

**Authors:** Shan Wang, Frederik-Jan van Schooten, Han Jin, Daisy Jonkers, Roger Godschalk

**Affiliations:** 1 Department of Pharmacology and Toxicology, School of Nutrition and Translational Research in Metabolism (NUTRIM), Maastricht University, 6200 MD Maastricht, The Netherlands; 2 Department of Pathology, School for Cardiovascular Diseases (CARIM), Maastricht University Medical Center+ (MUMC+), 6200 MD Maastricht, The Netherlands; 3 Department of Internal Medicine, Division Gastroenterology-Hepatology, School of Nutrition and Translational Research in Metabolism (NUTRIM), Maastricht University, 6200 MD Maastricht, The Netherlands

**Keywords:** inflammatory bowel disease, tryptophan metabolism, transcriptome, metabolome

## Abstract

Evidence is emerging for the role of intestinal tryptophan metabolism in the development of inflammatory bowel disease (IBD). In order to identify the role of altered intestinal tryptophan metabolism in IBD pathogenesis, a meta-analysis of the transcriptome was performed to identify differentially expressed genes involved in the tryptophan metabolism pathways in intestinal biopsies of IBD as compared to non-IBD controls. Moreover, a systematic review of the metabolome was performed to identify the concurrent changes in tryptophan metabolites. Integration of the transcriptome and metabolome identified various alterations in intestinal tryptophan metabolism during active disease in IBD patients, including decreased intestinal tryptophan absorption, enhanced kynurenine pathway, increased interstitial serotonin availability, changed indole pathway, and activated aryl hydrocarbon receptor signaling. Therefore, a network of intestinal tryptophan metabolism pathways in IBD could be established, helping to assess the potential of genes and metabolites involved in these pathways as diagnostic markers and targets for IBD management.

## 1. Introduction

Inflammatory bowel disease (IBD) includes two phenotypes: ulcerative colitis (UC) and Crohn’s disease (CD). UC is characterized by continuous inflammation that is limited to the colon, while CD involves any part of the gastrointestinal (GI) tract in a non-continuous fashion and, unlike UC, is commonly associated with complications, such as strictures, abscesses, and fistulas. Histologically, UC shows superficial inflammatory changes limited to the mucosa and submucosa, while CD can occur in all layers of the bowel wall [1]. With an alternating relapsing-remitting disease course, the outcome of both CD and UC could vary from minor symptoms with prolonged periods of remission to active disease with recurrent exacerbations and severe life-threatening conditions that result in hospitalization, surgical intervention, or even death [2]. Current treatment strategies aim at controlling mucosal inflammation, but these are not always effective. Patients in remission, even after surgical resection, often relapse [3]. So far, the etiology of IBD remains largely unknown, though it is hypothesized that the onset is due to an aberrant intestinal immune response to environmental triggers, catalyzed by the genetic susceptibility of the individual [4]. It is of great importance to gain a better understanding of IBD pathogenesis and expand the therapeutic armamentarium.

**Figure 1:**
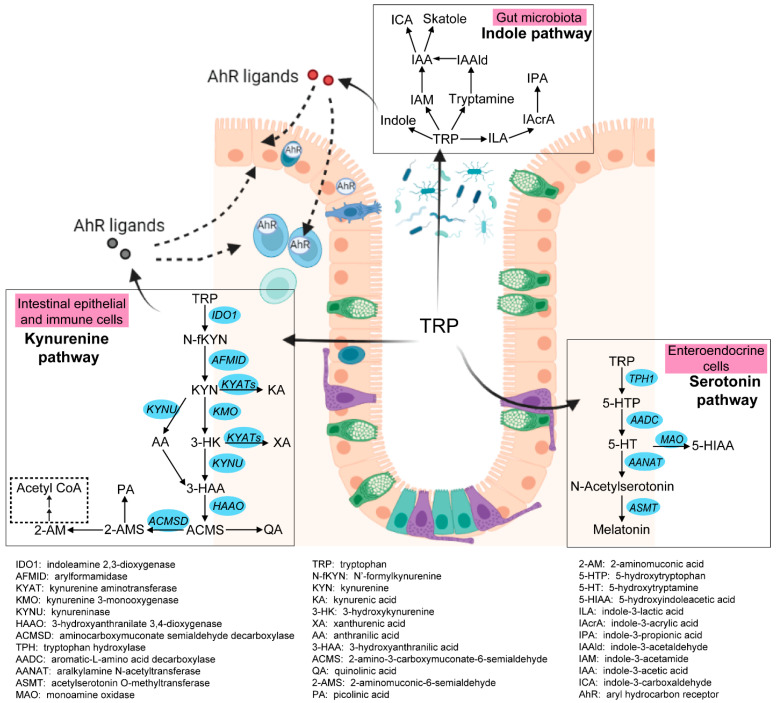
Three pathways of intestinal TRP metabolism. The genes involved in the kynurenine pathway and the serotonin pathway were indicated in blue. This figure was generated with BioRender (https://biorender.com/ (accessed on 29 September 2022)).

Intestinal tryptophan (TRP) metabolism involves a complex interaction between host genetic, microbial, and dietary factors. TRP is an essential amino acid that should be ingested via TRP-rich foods, such as lean meat, fish, dairy products, nuts and seeds, and so on [5]. TRP metabolism follows three main pathways in the GI tract [6,7] (see Figure 1). Firstly, the ingested TRP can be metabolized by the kynurenine pathway (KP) via the rate-limiting enzyme indoleamine 2,3-dioxygenase 1 (IDO1), with notable expression in mucosal and immune cells. Under normal physical conditions, an intestinal KP is present with a minimal (5–10%) contribution to TRP degradation, but this contribution may increase significantly after immune activation. Secondly, about 1–2% of dietary TRP enters the serotonin (5-hydroxytryptamine, 5-HT) pathway via tryptophan hydroxylase (TPH) in the gut, mainly within the enterochromaffin cells, generating approximately 95% of the total serotonin content of the human body. Thirdly, around 4–6% of TRP enters the indole pathway in gut microbiota, which produce a range of indole metabolites. These three pathways work separately but remain tightly interconnected in affecting gut homeostasis. Many TRP metabolites produced by these pathways were reported to affect the intestinal activation of aryl hydrocarbon receptor (AhR) signaling, which is important in modulating intestinal inflammation [8]. It is therefore worthwhile to study these pathways collectively to gain a complete and thorough understanding of the involvement of TRP metabolism in gut inflammatory disorders, most notably IBD.

Clinical and animal experiments have identified perturbations of TRP metabolism in the development of IBD. For instance, dietary TRP deficiency enhanced dextran sodium sulfate (DSS)-induced intestinal inflammation [9], while administration of TRP or TRP metabolites might ameliorate inflammation and regulate epithelial homeostasis [10,11,12]. Decreased serum levels of TRP and increased *IDO1* expression in mucosal samples were found in patients with IBD [13,14,15]. The severity of DSS-induced colitis was attenuated in *TPH1^−/−^* mice and in mice with inhibition of 5-HT synthesis, suggesting that 5-HT worsens intestinal inflammation [16]. These findings indicate that genes and metabolites involved in intestinal TRP metabolism could be potential biomarkers for intestinal inflammation and may be of interest for predicting relapse. In addition, modulation of intestinal TRP metabolism could offer potential targets for preventive and therapeutic interventions for IBD patients. So far, both TRP and its metabolite kynurenine (KYN), as well as the rate-limiting enzymes IDO1 and TPH1, have been widely studied in intestinal disorders, while the regulatory role of the other genes and metabolites within TRP metabolism pathways in IBD remains largely unexplored and thus needs to be investigated.

In order to gain a complete understanding of the three metabolic pathways of TRP in IBD, a meta-analysis of publicly available transcriptomics datasets derived from intestinal biopsies of IBD patients and non-IBD controls was performed to detect differentially expressed genes involved in the KP and serotonin pathways. Considering that these changes in gene expression might result in different concentrations of TRP and its metabolites in stool, blood, and intestinal biopsies; therefore, a systematic review of metabolomics studies was performed to identify how TRP and its metabolites changed in biological samples from IBD patients.

## 2. Materials and Methods

### 2.1. Meta-Analysis of the Transcriptome

**Figure 2:**
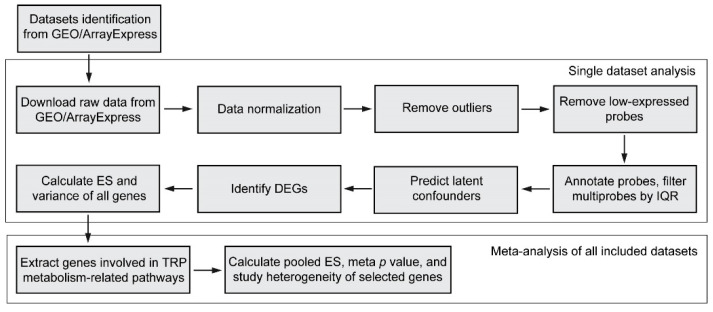
Summary of the meta-analysis pipeline applied in this study. IQR, interquartile range; DEGs, differentially expressed genes; ES, effect size.

The meta-analysis of the transcriptome pipeline consisted of (1) systematic review and dataset identification; (2) processing of a single transcriptomics dataset; and (3) meta-analysis of genes involved in TRP metabolism-related pathways across all datasets. The graphical summary of the workflow applied in this study is shown in Figure 2. Each step will be further explained in the following paragraphs.

#### 2.1.1. Systematic Review of the Transcriptome

The systematic review was performed following the Preferred Reporting Items for Systematic Reviews and Meta-Analyses (PRISMA) statement [17]. The search for the transcriptome was carried out in the NCBI’s Gene Expression Omnibus (GEO) and EMBL EBI’s ArrayExpress published between 1 January 2010 to 31 December 2021. The search terms “inflammatory bowel disease”, “Crohn’s disease”, and “ulcerative colitis” were combined. The search strategy included both searching Medical Subject Headings (MeSH) and free language words.

#### 2.1.2. Inclusion and Exclusion Criteria

Two investigators (SW and HJ) reviewed titles and abstracts independently. We obtained relevant datasets and evaluated them in more detail using pre-specified inclusion and exclusion criteria. The inclusion criteria were the following: (1) Gene expression profiles were generated with microarrays or high-throughput sequencing; (2) studies were performed on human samples; and (3) studies were performed on tissue samples. The exclusion criteria were: (1) studies were not involving IBD patients (either CD or UC); (2) tested samples were not intestinal biopsies; (3) the intestinal region of patients was not affected by the disease; (4) the unavailability of information on patient characteristics; (5) studies did not contain both diseased and suitable matched non-IBD controls in the same experimental batch; (6) gene expression was not measured by the Affymetrix, Illumina, or Agilent platforms; (7) sample sizes were smaller than 5 in each group; and (8) single-cell sequencing datasets were obtained. The disagreements between the two investigators were resolved through discussion.

#### 2.1.3. Processing of a Single Transcriptomics Dataset

All data analyses were performed in RStudio using R (version 4.1.1, Boston, MA, USA). The details of data processing specific to each study are available in Supplementary Materials. Effect sizes (ES) and variances were calculated for the meta-analysis. In this study, the ES of each gene for each study was measured as a between-group standardized mean difference (SMD), which is often called Cohen’s *d* value. A small-sample correction was further applied to SMD, which led to an ES called Hedges’ *g* value [18]. Briefly, this involves the calculation of Cohen’s *d* value (log2 fold change disease vs. control, divided by pooled standard deviation), followed by an adjustment of the number of arrays (known as *j* factors).

#### 2.1.4. Gene Selection

Genes involved in intestinal TRP metabolism-related pathways, including tryptophan absorption, the kynurenine pathway, the serotonin pathway, and AhR signaling, were identified according to the Kyoto Encyclopedia of Genes and Genomes (KEGG) pathway database, the Reactome Pathway Database, and previous publications. As a result, the expression levels of 42 genes (shown in Table 1) in the intestinal biopsies were subsequently compared between IBD patients and non-IBD controls.

#### 2.1.5. Meta-Analysis

A meta-analysis of the transcriptome was performed using the R package ‘meta’ [19]. In this study, the genes including *5-HTR_1A_*, *5-HTR_1C_*, *5-HTR_2C_*, *5-HTR_3D_*, *5-HTR_5A_*, *5-HTR_5B_*, *ASMT*, and *AHRR* that not detected in at least 80% of the included datasets were thereafter excluded for meta-analysis. For the 34 genes involved, pooled effect size (ES), standard error, *p* value, test of heterogeneity, and *I*^2^ were calculated using random effect models with the inverse variance method.

### 2.2. Systematic Review of the Metabolome

A comprehensive literature search was carried out in the database PubMed, focusing on publications between 1 January 2010 to 31 December 2021. Studies investigating the human metabolome in IBD were identified using specific search terms shown in Supplementary Materials. Then the titles and abstracts of full-text articles were screened by two investigators (SW and HJ) using the following eligibility criteria: (1) research article; (2) studies were performed on human samples; (3) studies were performed with IBD patients (either CD or UC); (4) metabolomics analysis was performed in bio-samples, including blood (either serum or plasma), stool, and/or intestinal biopsies. A full text review was carried out on the remaining papers following the exclusion criteria: (1) data were not compared between IBD and control; (2) other omics studies; and (3) tryptophan and/or its metabolites were not identified by the study.

## 3. Results and Discussion

### 3.1. Systematic Review of the Transcriptome and Metabolome

**Figure 3:**
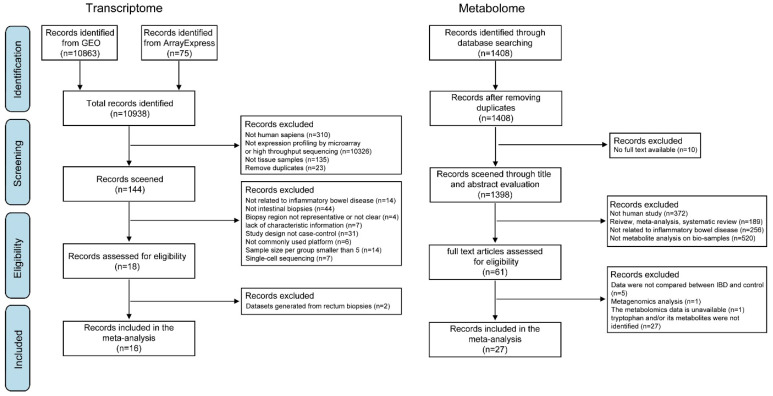
Flow diagram of the study selection process.

The selection strategies of both transcriptomics and metabolomics studies are presented in the PRISM diagram (Figure 3). For the transcriptome, a total of 10,938 datasets were identified, and 144 datasets were screened. After the application of inclusion and exclusion criteria, 18 datasets were eligible. Two datasets (GSE109142 and GSE114527) compared the gene expression profiles of rectal biopsies between IBD patients and controls. Only two rectal studies were insufficient for statistical analysis in the meta-analysis and were therefore excluded. Finally, 16 datasets (14 from GEO and 2 from ArrayExpress, Supplementary Table S1) were included. During data pre-processing, samples that failed to meet the data quality requirements were regarded as outliers and removed (see Supplementary Materials). We further subclassified all datasets according to IBD phenotypes (CD and UC), biopsy location (ileum and colon), and, when applicable, disease activity (inactive and active inflammation). As shown in Table 2, 8 datasets (592 iCD and 164 controls) were applied to compare gene expressions of ileal biopsies between CD and control, 6 datasets (163 cCD and 125 controls) to compare gene expressions of colonic biopsies between CD and control, and 9 datasets (406 cUC and 167 controls) to compare gene expressions of colonic biopsies between UC and controls.

For the metabolome, we identified 1408 studies for potential inclusion through the systematic literature review. After screening for titles and abstracts, 61 studies met the inclusion criteria. The full texts of the remaining studies were reviewed in detail, and of these, 27 studies were selected for inclusion. All studies identified for the review of tryptophan metabolism in biological samples of IBD are summarized in Table 3.

### 3.2. Meta-Analysis of Gene Expressions and Summary of Metabolites Involved in Intestinal TRP Metabolism

For the transcriptome, the log2 fold changes (LogFC) and statistical significances of involved genes compared between IBD and controls in each dataset are listed in Supplementary Table S2. There was no statistical significance in gene expression when compared between inactive IBD (either cCD, cUC, or iCD in inactive inflammation status) and controls in all included datasets. A meta-analysis of 34 genes compared between active IBD (either cCD, cUC, or iCD in active inflammation status) and controls was performed, and the pooled effect size (ES) and between-study heterogeneity of each gene are listed in Supplementary Table S3.

For the metabolome, the differences in TRP and its metabolites in bio-samples compared between IBD patients and controls were extracted from 27 selected metabolomics studies and summarized in Supplementary Table S4.

Integration of gene expression and metabolites within intestinal TRP metabolism pathways was described and discussed below.

#### 3.2.1. Decreased TRP Absorption in IBD Patients

**Figure 4:**
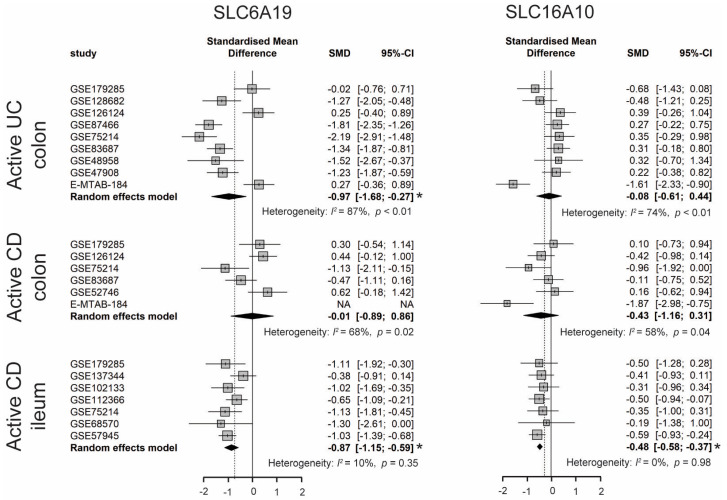
Forest plot for differential gene expression of *SLC6A19* and *SLC16A10* across studies of each IBD subtype as compared to non-IBD controls. * The *p*-value of the pooled effect size (ES) is less than 0.05. SMD, standardized mean difference; CI, confidence interval.

Dietary TRP is absorbed by enterocytes apically via the B^0^AT1 (encoded by *SLC6A19*) epithelial amino acid transporter and basolaterally transported via the aromatic amino acid transporter TAT1 (*SLC16A10*) protein [7]. As shown in Figure 4, the expression of *SLC6A19* was significantly lower in colonic biopsies of active UC and ileal biopsies of active CD. Moreover, the expression of *SLC16A10* was significantly decreased only in ileal biopsies of active CD. In accordance with decreased gene expression of TRP transporters, nine out of eleven studies identified decreased levels of TRP in the serum/plasma of patients with IBD, with a stronger reduction in patients with active disease. Additionally, five out of seven studies found that the TRP concentration increased in the stool samples of patients with IBD (Table S4). Even though the TRP consumption from the diet was unknown, these results suggest that IBD patients with active disease may have decreased TRP absorption from the intestinal tract.

#### 3.2.2. Enhanced Kynurenine Pathway (KP) in IBD Patients

**Figure 5:**
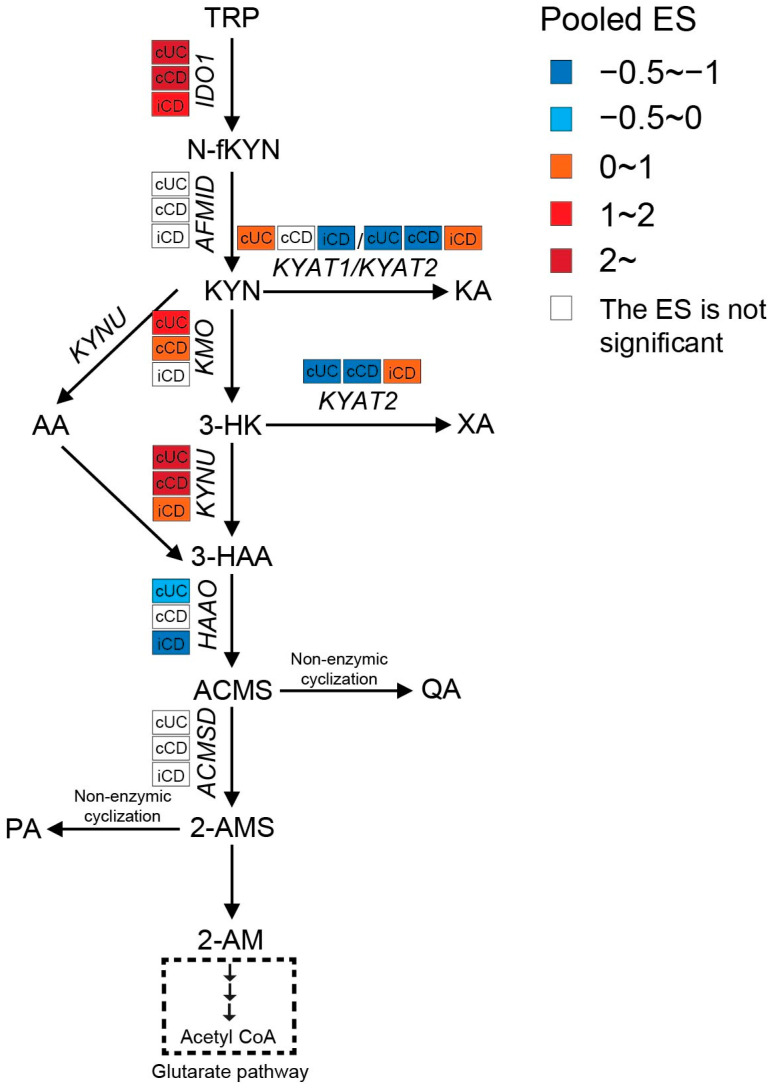
Pooled effect size (ES) for genes involved in KP across studies of each IBD subtype as compared to non-IBD controls. For an additional forest plot of these genes, see Supplementary Figure S1. cUC, colonic biopsies of active UC; cCD, colonic biopsies of active CD; iCD, ileal biopsies of active CD.

Intestinal TRP metabolism through KP is mediated by the rate-limiting enzyme IDO1, which results in the production of KYN. KYN is metabolized mainly by hydroxylation to 3-hydroxykynurenine (3-HK) by kynurenine 3-monooxygenase (KMO), followed by hydrolysis of 3-HK to 3-hydroxyanthranilic acid (3-HAA) by kynureninase (KYNU). As shown in Figure 5, the expression levels of *IDO1*, *KMO*, and *KYNU* were significantly higher in both active CD and UC patients, indicating an enhanced KP in the gut of IBD patients. Consistently, nine out of twelve metabolomics studies identified increased KYN and/or KYN/TRP levels in either blood, stool, or colonic biopsies of IBD, with an even stronger increase in patients with active inflammation (Table S4). The decreased TRP absorption (see Section 3.2.1) and enhanced KP may synergistically contribute to the reduced blood TRP levels in IBD (Table S4). When compared to inflamed colonic biopsies, the KP is less activated in inflamed ileal biopsies, as indicated by a smaller ES for *IDO1*, *KMO*, and *KYNU*.

Both KYN and 3-HK can be transaminated by kynurenine aminotransferases (KYATs) to form kynurenic acid (KA) and xanthurenic acid (XA). These reactions by KYATs are usually of minor significance because of the high *K*_m_ of their two substrates when compared to KMO and KYNU [45]. There are four KYAT isoenzymes reported, of which KYAT1 (also known as cysteine conjugate beta-lyase, CCBL1) and KYAT2 (aminoadipate aminotransferase, AADAT) play capital roles in humans. Although KYAT1 and KYAT2 possess overlapping biochemical properties, KYAT1 showed less catalytic efficiency but higher specific activity for the transformation of KYN to KA when compared to KYAT2. Moreover, KYAT1 is not actively involved in the transformation of 3-HK to XA [46]. A meta-analysis of the transcriptome showed significantly less expression of *KYAT1* in inflamed ileum and less *KYAT2* in inflamed colon of CD patients when compared to controls. Increased *KYAT1* and decreased *KYAT2* expressions were observed in colonic biopsies of UC patients. In line with gene expression, the metabolomics studies showed that serum levels of KA were lower in CD patients (especially in active status) in all included studies when compared to UC and controls, which could be a potential indicator of CD. XA also showed a decreasing trend in blood samples from IBD patients. It should be pointed out that *KYAT2* was induced in inflamed ileal biopsies but inhibited in colonic biopsies from CD patients. However, none of the metabolomics studies compared the XA levels between ileal-only CD and colon-only CD, which makes it difficult to validate its role in distinguishing these two subtypes. It is worthwhile to perform further studies to confirm the difference in *KYAT2* expression combined with XA concentration between ileal-only CD and colon-only CD. The differences in KA and XA between UC and controls are less clear.

The most active KP enzyme is 3-hydroxyanthranilate 3,4-dioxygenase (HAAO), which catalyzes the fast conversion of 3-HAA to 2-amino-3-carboxymuconate-6-semialdehyde (ACMS) and hence low 3-HAA levels in tissue and blood [47]. As shown in Figure 5, the gene expression of *HAAO* was significantly decreased in active cUC and iCD, with a stronger reduction in iCD compared to cUC. Enhanced expression of *IDO1* and *KYNU*, but inhibition of *HAAO* might result in accumulation of 3-HAA. Indeed, Huhn et al. [24] observed significantly elevated levels of 3-HAA specifically in inflamed ileal biopsies from CD patients, but not in colonic samples from CD or UC patients (Table S4), which might be a potential biomarker allowing the localization of the inflammation.

Subsequently, part of ACMS favors its non-enzymatic cyclization to quinolinic acid (QA). Studies with antibodies to QA demonstrated that immune cells such as mononuclear cells and tissue macrophages are the main cell types that are capable of synthesizing and storing QA. Based on previous findings, QA is strongly elevated when these cells are stimulated by immune activators such as IFN-γ [48]. Consistently, the systematic review of the metabolome also identified increased QA levels in blood, stool, and biopsy samples of IBD, especially in patients with active disease (Table S4). The other two metabolic branches involve decarboxylation of ACMS by 2-amino-3-carboxymuconate-6-semialdehyde decarboxylase (ACMSD) to form 2-aminomuconic-6-semialdehyde (2-AMS), which can further be converted to picolinic acid (PA) via non-enzymatic cyclization, to 2-aminomuconic acid (2-AM), and finally to acetyl coenzyme A (Acetyl-CoA). Acetyl-CoA feeds into the glutarate pathway to yield energy. Under physiological conditions, most TRP that enters the KP pathway is converted to ATP, CO_2_, and water in the glutarate pathway. PA is only produced when the flux of metabolites through the glutarate pathway is high and the enzymes of the glutarate pathway are saturated [49]. Our meta-analysis of the transcriptome showed no significant difference in *ACMSD* expression between IBD and controls (Figure 5). However, the metabolomics studies identified either decreased or unchanged PA levels in bio-samples of IBD compared to controls (Table S4). These results suggest enhanced activity of the glutarate pathway to refuel the energy deficiency of epithelial cells in inflamed intestinal biopsies from IBD patients.

KYN and most of its metabolites (including KA, XA, AA, 3-HK, 3-HAA, PA, and QA) are implicated in the regulation of the immune response. The production of KYN and its metabolites exert immunosuppressive effects mainly by inhibition of T cell function, activation of regulatory T cells, and inhibition of Natural Killer cells and antigen-presenting cells. These effects are, at least in part, mediated by the activation of the aryl hydrocarbon receptor (AhR), which has been shown to play an anti-inflammatory role in the immune response [50,51]. KA can also activate the orphan G protein-coupled receptor GPR35, which is predominantly detected in immune cells of the GI tract, to modulate intestinal inflammation [50]. The up-regulation of *IDO1*, *KMO*, and *KYNU* in intestinal biopsies, as well as increased levels of KYN in bio-samples of IBD patients, are therefore likely to be a negative feedback mechanism that suppresses the ongoing inflammatory response.

Intestinal TRP metabolism through the KP also yields neuroactive metabolites, among which KA and QA have been extensively studied. KA is a neuroprotective molecule that acts as an antagonist of the N-methyl-D-aspartate (NMDA), kainic acid, and alpha-amino-3-hydroxy-5-methyl-4-isoxazolepropionic acid receptors [52]. QA can exert neurotoxic activity by acting as an NMDA receptor agonist. The systematic review of the metabolome showed decreased blood KA concentrations in CD patients and increased blood QA levels in IBD patients. The decreased KA/QA could exert an excitotoxic effect on enteric neurons, which may be involved in intestinal hypermotility [53] and malfunction of gut-brain sensory transduction [54]. Moreover, the ratio of KA to QA in the blood was reduced in patients with major depressive disorders and was negatively correlated with blood C-reactive protein (CRP) level, one of the most commonly used measures of systemic inflammation in clinical practice [55,56]. These results may particularly provide a potential mechanism underlying the entero-active effect and the occurrence of depression in IBD patients.

#### 3.2.3. Increased Interstitial Serotonin Availability in IBD Patients

**Figure 6:**
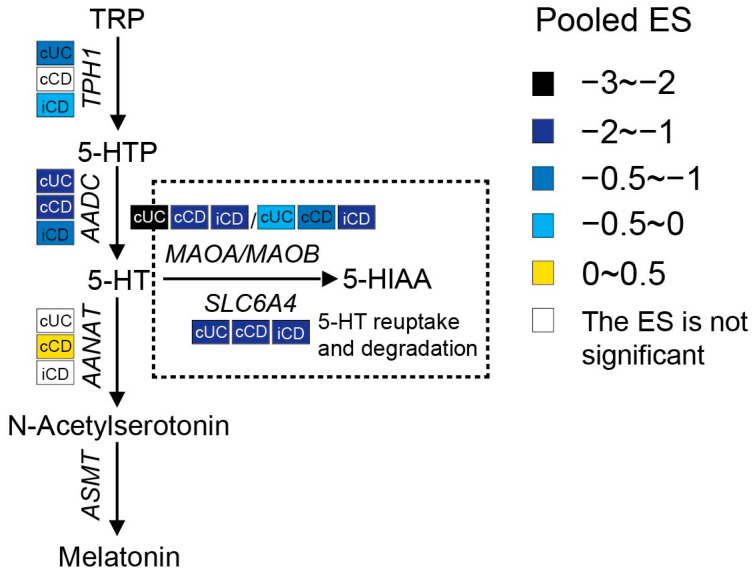
Pooled effect size (ES) for genes involved in the serotonin pathway across studies of each IBD subtype as compared to non-IBD controls. For additional forest plots of these genes, see Supplementary Figure S2. cUC, colonic biopsies of active UC; cCD, colonic biopsies of active CD; iCD, ileal biopsies of active CD.

Serotonin synthesis in enterochromaffin (EC) cells involves the rate-limiting step where TRP is converted to 5-hydroxytryptophan (5-HTP) by tryptophan hydroxylase 1 (TPH1), followed by decarboxylation to serotonin (5-HT) by aromatic-L-amino acid decarboxylase (AADC). As shown in Figure 6, a meta-analysis of the transcriptome showed that the expression of *TPH1* was significantly lower in inflamed colonic biopsies of UC patients but not in CD patients. Even though inflamed ileal biopsies from CD also expressed less *TPH1*, they had a smaller pooled ES. Gene expression levels of *AADC* were significantly decreased in both ileal and colonic biopsies of patients with active IBD, while an even stronger reduction was observed in UC patients. These results indicate the inhibition of serotonin synthesis in IBD patients with active inflammation. Colon tissue from UC patients showed stronger inhibition of the serotonin pathway when compared to CD patients.

Once synthesized, serotonin is rapidly packaged via the vesicular monoamine transporter into dense granules or vesicles located at the base of the cell. When EC cells are exposed to intraluminal pressure or chemical and mechanical stimulation, serotonin is released either apically to the gut lumen or basolaterally to the lamina propria. Upon its release, serotonin may take several possible routes, including having a direct influence on the gut microbiota, exerting influence on intracellular signaling by acting on 5-HT receptors (5-HTRs), or being reuptook by surrounding epithelial and immune cells via the serotonin reuptake transporter (SERT, encoded by the *SLC6A4* gene), or entering the blood. In blood, serotonin is present as free serotonin or it is taken up by platelets via SERT (approximately 95%) [57,58]. Any excess serotonin in the cells is degraded by monoamine oxidase (MAO), resulting in the production of 5-hydroxyindoleacetic acid (5-HIAA), which is mainly excreted in urine. There are two forms of MAO known: MAOA and MAOB. MAOA has the highest affinity for serotonin [7]. A meta-analysis of the transcriptome showed significantly lower levels of *SLC6A4* and *MAO* in all IBD patients with active inflammation, implying reduced reuptake and inhibited degradation of serotonin in intestinal tissues from IBD patients.

Serotonin can also be shunted into the melatonin pathways. Extra-pineal melatonin synthesis can occur in the EC cells, where serotonin can be metabolized to N-acetylserotonin by aralkylamine N-acetyltransferase (AANAT) and eventually to melatonin by acetylserotonin O-methyltransferase (ASMT). There was no obvious change in *AANAT* expression between IBD and controls. Moreover, *ASMT* was not detected in at least 80% of the included datasets, which represents the relatively low melatonin synthesis in the gut.

For the metabolites produced during this pathway (shown as Table S4), the serum level of 5-HTP was decreased in IBD identified by two studies, which is in line with reduced expression of *TPH1*. There was no significant difference in stool serotonin levels between IBD and control. However, four out of six studies identified increased serotonin levels in blood samples of IBD patients as compared to controls or in active CD patients as compared to inactive CD patients. Taken the transcriptome and metabolome together, these results suggest that there is an increased interstitial availability of serotonin in the lamina propria, leading to successive activation of a variety of 5-HTRs present on smooth muscles, enteric neurons, enterocytes, and immune cells to exert its biological function.

There are seven 5-HTR families identified so far, and five (5-HTR_1_, 5-HTR_2_, 5-HTR_3_, 5-HTR_4_, 5-HTR_7_) are expressed in the gut [59]. The conventional effects of serotonin in the gut and responding mediated receptors were summarized by Liu et al. [60] and Mawe et al. [61]. By activating these 5-HTRs, serotonin plays an important role in regulating motility, secretion, and sensory function in the gut. Furthermore, accumulating clinical and animal studies have identified the immunomodulatory role of serotonin in intestinal inflammation. Several 5-HTRs were reported to be expressed on immune cells, including B and T lymphocytes, monocytes, macrophages, and dendritic cells [62]. Through activating 5-HTRs during intestinal inflammation, serotonin can either serve as a pro-inflammatory mediator [16,63] or exert an anti-inflammatory effect on intestinal mucosa [64]. In the present study, a meta-analysis of the transcriptome showed that gene expression levels of 5-HTR_3C_ and 5-HTR_3E_ were significantly lower in inflamed biopsies from both CD and UC (Table S3). Li et al. identified that the decreased expression of 5-HTR was the dominant mechanism underlying the desensitization of 5-HTR observed in SERT^−/−^ mice [65]. The consistent lower levels of *5-HTR_3C_* and *5-HTR_3E_* among both IBD phenotypes suggests the desensitization of these receptors in response to successive potentiation of serotonin, which may reflect protective mechanisms. Additionally, the downregulation of *5-HTR_1D_* and *5-HTR_3A_* as well as the upregulation of *5-HTR_2B_* were observed only in inflamed ileal biopsies from CD, and the decreased *5-HTR_1E_* and *5-HTR_4_* and increased *5-HTR_2A_* expression were specific to colonic biopsies from UC patients. The difference in 5-HTR dysregulation between IBD subtypes could at least partially explain the different clinical symptoms; it would be a potential diagnostic and therapeutic tool to improve the differentiation between IBD subtypes and, accordingly, to achieve better management of the disease. So far, there is limited research that systematically describes the 5-HTRs within the gut wall in the setting of IBD. Further human studies are required to completely understand the pathophysiological mechanisms of IBD underlying serotonin and its receptors.

#### 3.2.4. Altered Indole Pathway in IBD Patients

TRP that is not taken up by the ‘upper’ GI tract can be metabolized by resident microbes through the indole pathway, leading to the production of various indole metabolites (Figure 1). The bacterial species involved in the indole pathway have been summarized in several recent reviews [66,67]. Lines of studies have revealed that these indole metabolites generated by gut microbiota are important signaling molecules in microbial communities and host-microbial cross-talk and therefore influence host immunity and gut homeostasis. Indole is an interspecies and intraspecies signaling molecule of microbial communities that is important in modulating antibiotic resistance, plasmid stability, virulence, sporulation, and biofilm formation [68]. Moreover, in vitro cell experiments identified the regulatory role of indole in the secretion of glucagon-like peptide-1 (GLP-1) from intestinal enteroendocrine L cells, which is involved in stimulating insulin secretion, suppressing appetite, and slowing gastric emptying [69]. Tryptamine, an indole metabolite produced by *Ruminococcus gnavus* and *Clostridium sporogene* [70], was found to stimulate intestinal secretion via activation of 5-HTR_4_ [71,72]. IPA can regulate intestinal barrier integrity through the pregnane X receptor [73]. Moreover, several indole metabolites exert protective effects in maintaining intestinal barrier function and regulating immune responses during intestinal inflammation via binding to the aryl hydrocarbon receptor (AhR), which is present in different cell types of the intestinal mucosa (details are described in Section 3.2.5) [67].

The systematic review of the metabolome only identified a limited number of studies comparing indole metabolites in biosamples between IBD and controls. Two out of three studies found that indole levels were increased in stool samples from CD. Most studies showed that IAcrA and IPA levels decreased in either serum or stool samples obtained from active IBD patients. However, there are discrepancies in findings regarding tryptamine, IAA, and I3M levels between different biological samples (i.e., serum and stool samples) and between different studies. The alterations in these indole metabolites nevertheless indicated the changes in composition and diversity of the intestinal microorganisms in IBD pathogenesis. The contributions of these microorganisms responsible for TRP metabolism as well as the derived indole metabolites to gut health remain to a large extent unknown, which needs to be investigated in the future.

#### 3.2.5. Activated AhR Signaling in IBD Patients

Tryptophan metabolites from all three metabolic pathways, including KYN, KA, XA, serotonin, and microbiome-derived tryptophan metabolites, were reported to be AhR ligands and/or human AhR selective modulators [74,75,76]. The AhR is a ligand-activated transcription factor of the basic helix-loop/per ARNT-Sim (bHLH/PAS) superfamily. With ligand binding, AhR translocates from the cytosol into the nucleus, where the AhR heterodimerizes with the aryl hydrocarbon receptor nuclear translocator (ARNT). The AhR-ARNT complex then binds to dioxin-responsive elements or xenobiotic-responsive elements in the promoter of a battery of genes including *CYP1A1*, *CYP1B1*, *AHRR*, and *IL-22*, and induces their expression [77]. Previous studies showed enhanced DSS-induced intestinal inflammation in AhR^−/−^ mice [78], while AhR activation by supplementation of TRP or its metabolites ameliorated inflammation progression [9,12]. These results demonstrate that intestinal TRP metabolism can modulate gut homeostasis via AhR. Indeed, AhR activation in intestinal epithelial cells (IECs) controls cell renewal and turnover, induces expression of IL-10 receptors, and tight junctions, preserving intestinal barrier integrity and regulating tissue regeneration [79]. AhR signaling is also essential for the development, maintenance, and function of antigen-presenting cells, Th17/Th22 cells, innate lymphoid cells (ILCs), and intraepithelial lymphocyte γδ T cells (IELs), which are involved in modulating intestinal immunity [80]. IL-22, produced by ILCs, IELs, and Th17/Th22 cells via an AhR-dependent manner, has been shown to induce the production of antimicrobial peptides and mucins by IECs, limiting the pathogenicity and invasiveness of intestinal microorganisms.

**Figure 7:**
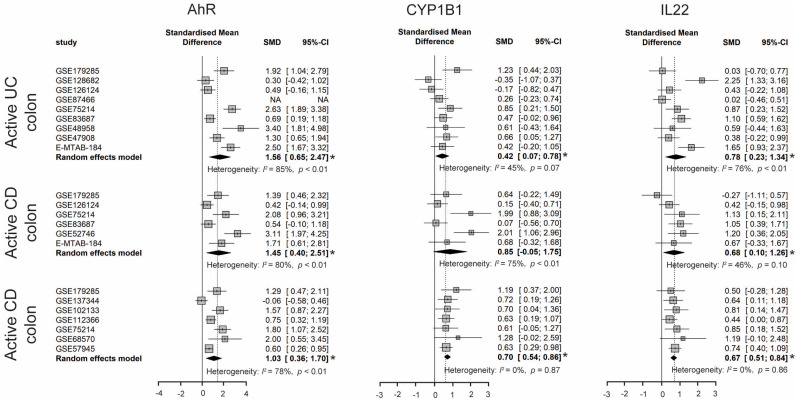
Forest plot for differential gene expression of *AhR*, *CYP1B1*, and *IL22* across studies of each IBD subtype as compared to non-IBD controls. * The *p*-value for ES is less than 0.05. SMD, standardized mean difference; CI, confidence interval.

A meta-analysis of the transcriptome showed that the expression levels of *AhR*, *CYP1B1*, and *IL-22* were significantly increased in the inflamed biopsies from all IBD patients when compared to biopsies from control subjects, suggesting activated AhR signaling in the inflamed intestine of IBD (Figure 7). However, the downstream gene of AhR, *CYP1A1*, was not changed in IBD. The suppression of *CYP1A1* induction might be due to a mutually antagonistic interaction of AhR with NF-κB in a gene-specific manner under inflammatory conditions (reviewed by Vondracek et al. [81]).

In line with our findings from the meta-analysis, Nikolaus et al. also identified higher serum IL-22 levels in IBD patients with active disease when compared to inactive IBD patients [13]. Additionally, AhR expression was upregulated in the lamina propria of CD patients, as determined by immunofluorescence staining, when compared to control biopsies [82]. However, these results conflict with some previous studies. Monteleone et al. found significantly decreased AhR mRNA and protein levels in mucosal biopsies of CD patients when compared to healthy controls and UC patients. Analysis of lamina propria mononuclear cells showed AhR expression was diminished in CD, particularly in CD3^+^, CD4^+^, CD56^+^, and CD25^+^ cells [83]. One study also identified reduced AhR activation ability of fecal water from IBD patients as compared to fecal water from healthy controls; therefore, reduced AhR activation of intestinal biopsies can be assumed [37]. The reason for these discrepancies is yet unknown, but it might result from (1) different sample sizes; (2) lifestyles (such as smoking and dietary tryptophan intake) and medications that might affect intestinal AhR activation were not considered in the comparison; and (3) unknown AhR ligands in the gut lumen that influence the AhR activation of intestinal biopsies. It still needs to be explored in the future whether AhR activation is the result of intestinal inflammation or whether AhR deficiency is the cause of intestinal inflammation.

## 4. Conclusions

Integration of the transcriptome and metabolome showed that each pathway of intestinal TRP metabolism was affected in the pathogenesis of IBD, especially in active disease, but remained tightly interconnected with each other. Based on these findings, detecting TRP metabolites in biological samples could be a potential marker to indicate active intestinal inflammation. Aberrant production of neuroactive metabolites such as KA, QA, and serotonin could explain the underlying mechanism of IBD-related psychological distress. Moreover, there will be potential strategies in IBD management through regulating intestinal TRP metabolism by: (1) modulating the enzymes that are involved in intestinal TRP metabolism; (2) regulating the binding of TRP metabolites to their receptors (e.g., 5-HTRs and AhR); and (3) supplementing probiotics or prebiotics to adjust gut microbiota. Further investigation is required with in-depth knowledge of the regulatory mechanisms and host-microbiota interaction to define the appropriate intervention for IBD patients and to have the ability to precisely act on the targeted metabolite or enzyme.

## Figures and Tables

**Table 1:** Genes selected for the meta-analysis on the basis of their gene function and involvement in relevant pathways.

Gene Function	Gene Symbols
Tryptophan absorption	*SLC6A19*, *SLC16A10*
Kynurenine pathway	*IDO1*, *AFMID*, *KYAT1*, *KYAT2*, *KMO*, *KYNU*, *HAAO*, *ACMSD*
Melatonin biosynthesis	*TPH1*, *AADC*, *AANAT*, *ASMT* *
Serotonin transporter	*SLC6A4*
Serotonin metabolism enzyme	*MAOA*, *MAOB*
Serotonin (5-HT) receptors	*5-HTR_1A_* *, *5-HTR_1B_*, *5-HTR_1C_* *, *5-HTR_1D_*, *5-HTR_1E_*, *5-HTR_1F_*, *5-HTR_2A_*, *5-HTR_2B_*, *5-HTR_2C_* *, *5-HTR_3A_*, *5-HTR_3B_*, *5-HTR_3C_*, *5-HTR_3D_* *, *5-HTR_3E_*, *5-HTR_4_*, *5-HTR_5A_* *, *5-HTR_5B_* *, *5-HTR_6_*, *5-HTR_7_*
AhR signaling pathway	*AhR*, *ARNT*, *AHRR* *, *CYP1A1*, *CYP1B1*, *IL22*

* These genes, which were not detected in at least 80% of the included transcriptomics datasets, were excluded for meta-analysis.

**Table 2:** Characteristics of transcriptomics datasets selected for meta-analysis.

GEO ID/ ArrayExpress ID (Publication Year)	Sample Size after Removing Outliers		Inflammation Status of IBD Patients		Platform	Adult/ Pediatric Cohort
	Disease	Control	Active	Inactive		
CD vs. Control (ileum)						
GSE179285 (2021)	62	8	33	29	Agilent-014850 Whole Human Genome Microarray 4 × 44k G4112F (GPL6480)	Adult
GSE137344 (2020)	99	29	28	71	NextSeq 550 (GPL21697)	Pediatric
GSE102133 (2019)	54	11	54	0	Affymetrix human gene 1.0 ST Array (GPL6244)	Adult
GSE112366 (2019)	105	26	105	0	Affymetrix HT HG-U133 + PM Array Plate (GPL13158)	Adult
E-MTAB-5790 (2018)	36	32	0	36	Agilent Whole Human Genome 4 × 44k Microarray	Pediatric
GSE75214 (2017)	67	11	51	16	Affymetrix Human Gene 1.0 ST Array (GPL6244)	Adult
GSE68570 (2016)	6	5	6	0	Illumina HumanHT-12 V4.0 expression BeadChip (GPL10558)	Adult
GSE57945 (2014)	163	42	163 #	0	Illumina HiSeq 2000 (GPL11154)	Pediatric
CD vs. Control (colon)						
GSE179285 (2021)	71	11	11	60	Agilent-014850 Whole Human Genome Microarray 4 × 44k G4112F (GPL6480)	Adult
GSE126124 (2019)	38	19	38 #	0	Affymetrix human gene 1.0 ST Array (GPL6244)	Pediatric
GSE75214 (2017)	8	11	8	0	Affymetrix Human Gene 1.0 ST Array (GPL6244)	Adult
GSE83687 (2017)	12	48	12	0	Illumina HiSeq 2500 (GPL16791)	Pediatric and adult
GSE52746 (2014)	10	17	10	0	Affymetrix Human Genome U133 Plus 2.0 Array (GPL570)	Adult
E-MTAB-184 (2012)	24	19	5	19	Illumina HumanHT-12 v3.0 Expression BeadChip	Adult
UC vs. Control (colon)						
GSE179285 (2021)	40	11	20	20	Agilent-014850 Whole Human Genome Microarray 4 × 44k G4112F (GPL6480)	Adult
GSE128682 (2020)	28	16	14	14	NextSeq 550 (GPL21697)	Adult
GSE126124 (2019)	18	19	18 #	0	Affymetrix human gene 1.0 ST Array (GPL6244)	Pediatric
GSE87466 (2018)	86	20	86 #	0	Affymetrix HT HG-U133 + PM Array Plate (GPL13158)	Adult
GSE75214 (2017)	97	11	74	23	Affymetrix Human Gene 1.0 ST Array (GPL6244)	Adult
GSE83687 (2017)	25	48	25	0	Illumina HiSeq 2500 (GPL16791)	Pediatric and adult
GSE48958 (2015)	13	8	7	6	Affymetrix human gene 1.0 ST Array (GPL6244)	Adult
GSE47908 (2014)	38	15	38	0	Affymetrix Human Genome U133 Plus 2.0 Array (GPL570)	Adult
E-MTAB-184 (2012)	61	19	21	40	Illumina HumanHT-12 v3.0 Expression BeadChip	Adult

# The activity information was not mentioned in the dataset or article. However, principal component analysis indicated that the first two principal components showed strong separation of disease groups from control (while the inactive patients could not). All samples were processed as active disease.

**Table 3:** Characteristics of metabolomics studies included in the systematic review.

Study (Publication Year)	Patients with CD		Patients with UC		Control	Biosample	Analytical Technique
	Active	Inactive	Active	Inactive			
Di’Narzo et al. (2022) [20]	284 *		360		329	Serum	LC-MS
	88		101		72	Stool	
Gu et al. (2021) [21]	/	/	93	/	102	Serum	LC-MS
Wang et al. (2021) [22]	29	/	/	/	20	Stool	LC-MS
Notararigo et al. (2021) [23]	/	18	/	9	10	Serum	^1^H-NMR
Huhn et al. (2020) [24]	12	/	11	/	12	Intestinal biopsy	Immunohistochemistry
Manzella et al. (2020) [25]	15	15	15	15	/	Serum	LC-MS
Diab et al. (2019) [26]	/	/	18	10	14	Intestinal biopsy	GC-MS LC-MS
Franzosa et al. (2019) [27]	68		53		34	Stool	LC-MS
Lai et al. (2019) [28]	10	10	/	/	10	Serum	LC-MS
Lloyd-Price et al. (2019) [29]	67		38		27	Stool	LC-MS
Whiley et al. (2019) [30]	/	/	19		10	Plasma	LC-MS
Shajib et al. (2019) [31]	21	19	/	/	40	Plasma	ELISA
Alexeev et al. (2018) [32]	/	/	15	20	20	Serum	HPLC-electrochemical coulometric array
Bosch et al. (2018) [33]	15	/	15	/	15	Stool	HPLC-UV
Scoville et al. (2018) [34]	8	12	19	1	20	Serum	LC-MS
Abautret-Daly et al. (2017) [35]	8		10		19	Plasma	HPLC coupled PDA-UV and fluorescence detectors
Kolho et al. (2017) [36]	36	/	20	/	29	Serum and stool	LC-MS
Nikolaus et al. (2017) [13]	81		67		100	Serum	LC-MS
Lamas et al. (2016) [37]	54 #				32	Stool	HPLC-coulometric electrode assay LC-MS
Yu et al. (2016) [38]	/	/	/	33	30	Plasma	HPLC-electrochemical detector
De Preter et al. (2015) [39]	29	54	28	40	40	Stool	GC-MS
Kohashi et al. (2014) [40]	/	/	52	68	120	Serum	GC-MS
Yau et al. (2014) [41]	15	10	14	5	9	Plasma	GC-MS
Walton et al. (2013) [42]	22	/	20	/	19	Stool	GC-MS
Hisamatsu et al. (2012) [43]	29	73	38	64	102	Plasma	HPLC-spectrophotometry
Gupta et al. (2012) [15]	20	5	/	/	11	Serum	HPLC coupled UV-V detector
Ooi et al. (2011) [44]	16	5	1	12	17	Serum	GC-MS

* The number of active and inactive patients was not indicated in the paper; # the number of CD and UC patients was not indicated in the paper; all 54 IBD patients were in remission.

## Data Availability

All transcriptome datasets analyzed in this study are publicly available in the NCBI’s Gene Expression Omnibus (GEO) and EMBL EBI’s ArrayExpress database. All metabolome data underlying this study are available in the article and in its online supplementary materials.

## Supplementary Materials

The following supporting information can be downloaded at: https://www.mdpi.com/article/10.3390/nu15132886/s1, Supplementary Methods: “Processing of single transcriptomic dataset” and “Search strategy for systematic review of metabolomics”; Table S1: Characteristics of transcriptomics datasets selected for meta-analysis; Table S2: Summary of differentially expressed genes compared between IBD and controls; Table S3: Meta-analysis of 34 genes compared between active IBD and controls; Table S4: Comparison of tryptophan and its metabolites in bio-samples derived from IBD patients and controls; Supplementary figures: Forest plots for differential gene expressions of kynurenine and serotonin pathways across studies of each IBD subtype as compared to non-IBD controls.

## Abbreviations

2-AM: 2-aminomuconic acid; 2-AMS: 2-aminomuconic-6-semialdehyde; 3-HAA: 3-hydroxyanthranilic acid; 3-HK: 3-hydroxykynurenine; 5-HIAA: 5-hydroxyindoleacetic acid; 5-HT: 5-hydroxytryptamine; 5-HTTP: 5-hydroxytryptophan; 5-HTRs: 5-hydroxytryptamine receptors; AA, anthranilic acid; AADC: aromatic-L-amino acid decarboxylase; AANAT: aralkylamine N-acetyltransferase; ACMS: 2-amino-3-carboxymuconate-6-semialdehyde; ACMSD: 2-amino-3-carboxymuconate-6-semialdehyde decarboxylase; Acetyl-CoA: acetyl coenzyme A; AFMID: arylformamidase; AhR: aryl hydrocarbon receptor; ASMT: acetylserotonin O-methyltransferase; AHRR: aryl hydrocarbon receptor repressor; ARNT: aryl hydrocarbon receptor nuclear translocator; CD: Crohn’s disease; CYP1A1: cytochrome P450 family 1 subfamily A member 1; CYP1B1: cytochrome P450 family 1 subfamily B member 1; DSS: dextran sodium sulfate; EC: enterochromaffin; ES: effect size; GI: gastrointestinal; HAAO: 3-hydroxyanthranilate 3,4-dioxygenase; IAA: indole-3-acetic acid; IAAld: indole-3-acetaldehyde; IAcrA: indole-3-acrylic acid; IAM: indole-3-acetamide; IBD: inflammatory bowel disease; ICA: indole-3-carboxaldehyde; IDO: indoleamine 2,3-dioxygenase; IECs: intestinal epithelial cells; IELs: intraepithelial lymphocytes; IL22: interleukin 22; ILA: indole-3-lactic acid; ILCs: innate lymphoid cells; IPA: indole-3-propionic acid; KA: kynurenic acid; KMO: kynurenine 3-monooxygenase; KP: kynurenine pathway; KYATs: kynurenine aminotransferases; KYN: kynurenine; KYNU: kynureninase; MAO: monoamine oxidase; PA: picolinic acid; NMDA: N-methyl-D-aspartate; QA: quinolinic acid; SLC6A4: solute carrier family 6 member 4; SLC6A19: solute carrier family 6 member 19; SLC16A10: solute carrier family 16 member 10; TPH, tryptophan hydroxylase; TRP, tryptophan; UC: ulcerative colitis; XA: xanthurenic acid.
