# Supplementary material for: The Involvement of Intestinal Tryptophan Metabolism in Inflammatory Bowel Disease Identified by a Meta-Analysis of the Transcriptome and a Systematic Review of the Metabolome
Source: Nutrients. 2023 Jun 26;15(13):2886. doi: 10.3390/nu15132886 (PMC10346271; doi:10.3390/nu15132886)
Supplement: Supplementary file 1 [file nutrients-15-02886-s001.zip › supplementary data/Supplementary figures.docx]

**
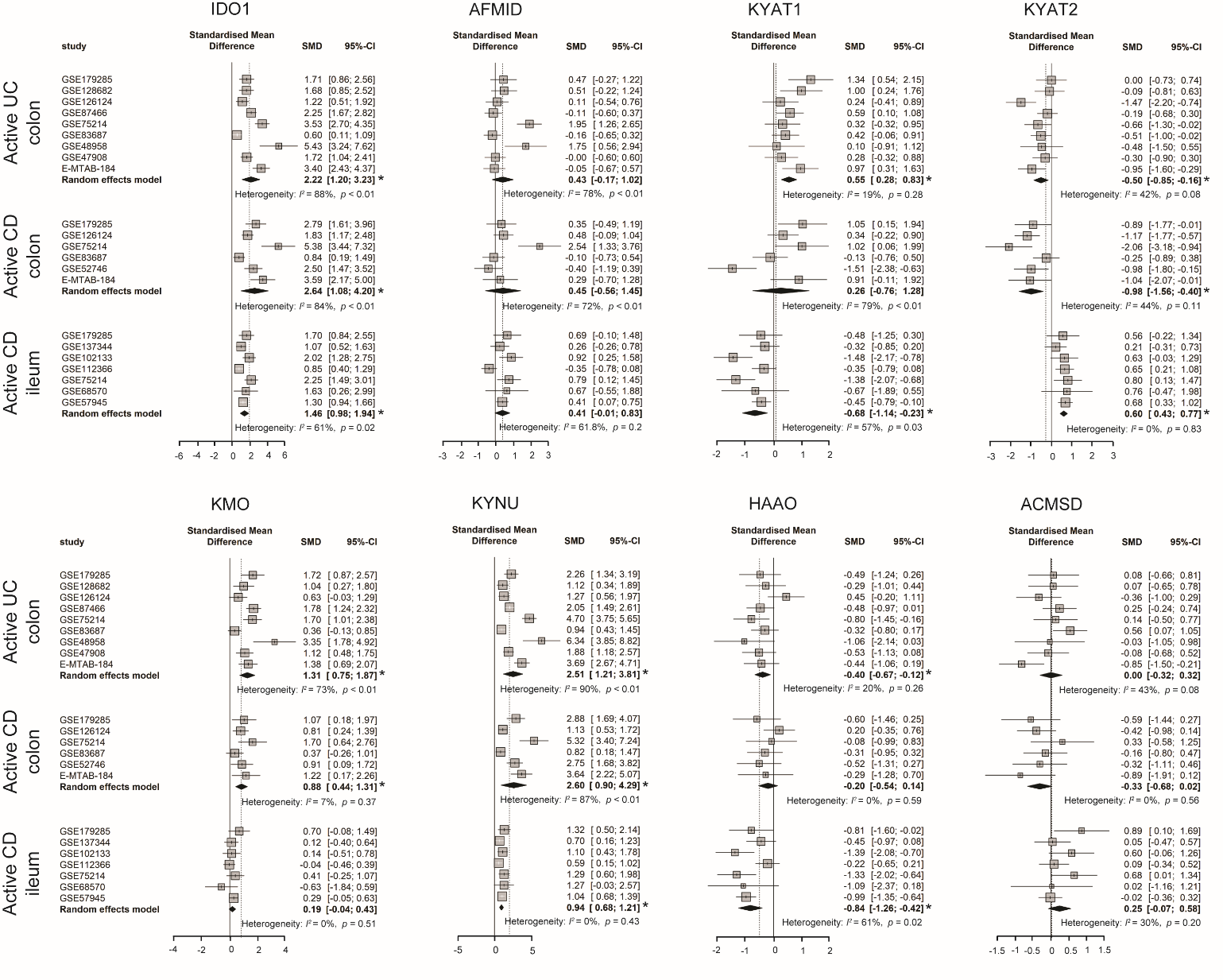
Figure S1.** Forest plots for differential gene expressions of kynurenine pathway across studies of each IBD subtype as compared to non-IBD controls. *, the *p* value for effect size (ES) is less than 0.05. SMD, standardized mean difference; CI, confidence interval.
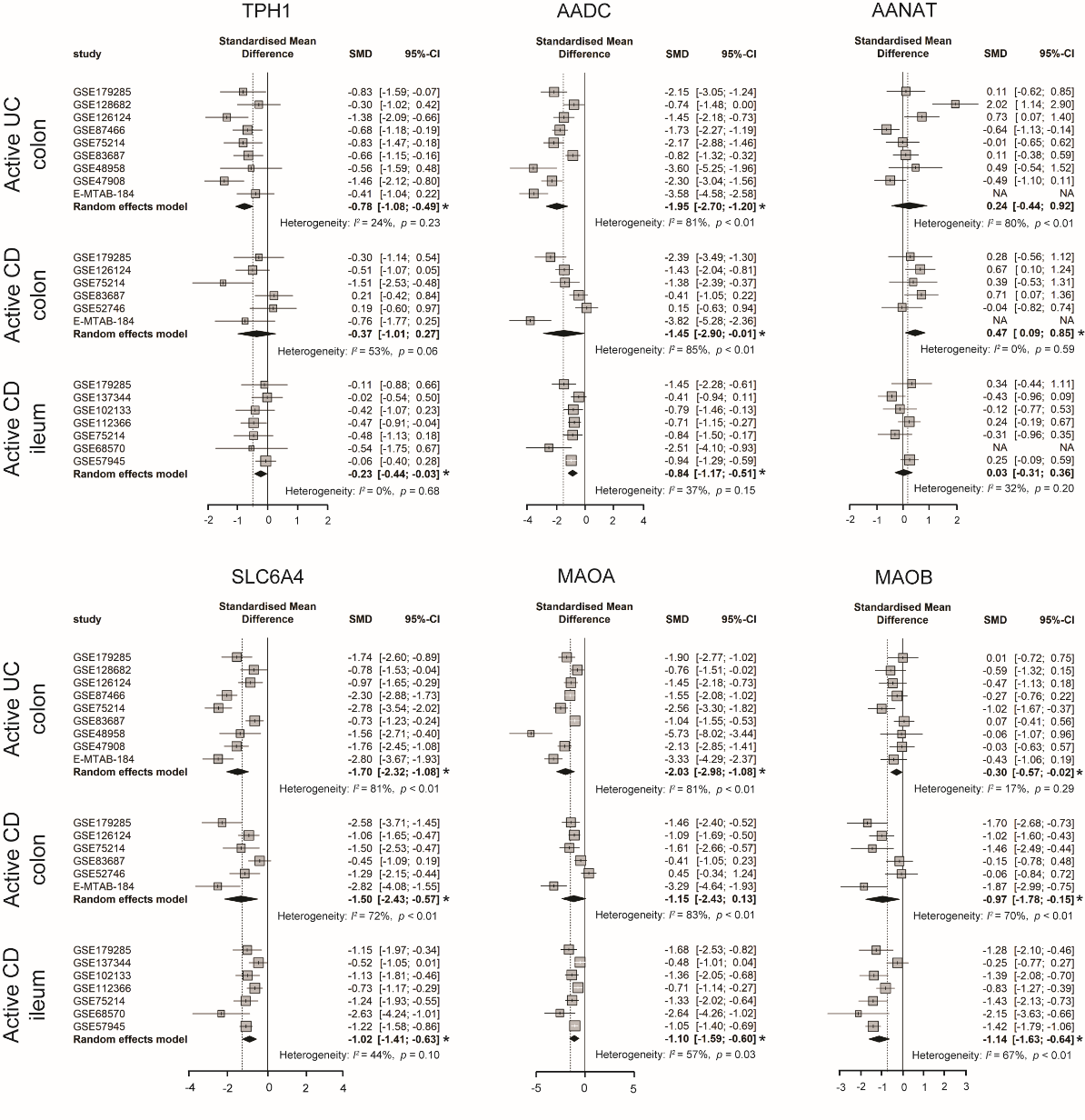


**Figure S2.** Forest plots for differential gene expressions of serotonin pathway across studies of each IBD subtype as compared to non-IBD controls. *, the *p* value for ES is less than 0.05. SMD, standardized mean difference; CI, confidence interval.
