# Supplementary material for: The Involvement of Intestinal Tryptophan Metabolism in Inflammatory Bowel Disease Identified by a Meta-Analysis of the Transcriptome and a Systematic Review of the Metabolome
Source: Nutrients. 2023 Jun 26;15(13):2886. doi: 10.3390/nu15132886 (PMC10346271; doi:10.3390/nu15132886)
Supplement: Supplementary file 1 [file nutrients-15-02886-s001.zip › supplementary data/supplementary methods.docx]

**Supplementary Methods**

1. Processing of single transcriptomic dataset

Transcriptomic data were downloaded either from GEO or ArrayExpress database. Data pre-processing was performed according to the platform of each study:

1. Affymetrix platform: Raw data were processed with the Robust Multi-array Average (RMA) algorithm through the “oligo” R package.^1^ Data quality was assessed via the arrayQualityMetrics package.^2^ Accordingly, outliers were identified and removed. Probes below the 10th percentile of the expression scale in over 80% samples were regarded as “unexpressed probes” and were excluded from further analysis. Probe to gene symbol annotation was done through specific annotation packages for each chip model available in the Bioconductor repository. Probes that map to multiple gene symbols were excluded from analysis. When multiple probes mapped to the same gene, the probe with greatest interquartile range (IQR) across samples was selected.
2. Agilent platform: Raw data were processed by using the “limma” package.^3^ For dataset GSE179285 (two-color microarray), data was processed as background correction, within-array normalization and between-array normalization. For dataset E-MTAB-5790 (single-channel microarray), data was processed as background correction and between-array normalization. Quality assessment was based on the evaluation of MA-plots, background intensities boxplot, and multi-dimensional scaling (MDS) plot, as recommended by limma user’s guide. Extremely deviant samples in any of the metrics were excluded. For single-channel Agilent microarray data, probes that are expressed above background in at least one sample per group (either diseased or control) were retained. Probes to gene symbol annotation were made from tables provided by the company responsible for the platform. Control probes and probes without symbols were excluded from analysis. When multiple probes mapped to the same gene, the probe with greatest interquartile range (IQR) across samples was selected.
3. Illumina BeadChip: Raw data were analyzed using “limma” package. Data was processed as background correction and normalization using neqc function. Quality assessment was based on the MDS plot, as recommended by limma user’s guide. Probes with a detection p-value >0.1 in all samples were regarded as “unexpressed probes” and were excluded.
4. RNAseq: Raw counts data were processed using “edgeR” package.^4^ Count per million (CPM) above 0 in at least 3 samples per group (either disease or control) were included for downstream analysis. The calcNormFactors function was applied to normalize the library sizes by calculating trimmed mean of M-values (TMM). Quality assessment was based on the MDS plot, as recommended by limma user’s guide.

Following data pre-processing, the Surrogate Variable Analysis was performed to estimate and remove potent confounders by using R package “sva”.^5^ Differentially expressed genes (DEGs) were identified via “limma” package.

2. Search strategy for systematic review of metabolomics: ① AND ② AND ③

① (((inflammatory bowel disease[MeSH Terms]) OR (inflammatory bowel disease)) OR (ulcerative colitis)) OR (Crohn's disease)

② (((((((((((metabolomics) OR (metabolome)) OR (metabolite)) OR (metabolic profile)) OR (biomarker)) OR (tryptophan metabolism)) OR (tryptophan)) OR (tryptophan metabolite)) OR (tryptophan catabolism)) OR (kynurenine)) OR (serotonin)) OR (indole)

③ (((((((((multiomic) OR (profiling)) OR (exp mass spectrometry/)) OR (exp nuclear magnetic resonance/)) OR (exp spectroscopy/)) OR (exp spectrophotometry/)) OR (LC-MS)) OR (GC-MS)) OR (NMR)) OR (HPLC)

References

1. 1. Carvalho B S,Irizarry R A. A framework for oligonucleotide microarray preprocessing*.* *Bioinformatics* 2010; **26**(19): 2363-2367.
2. 2. Kauffmann A, Gentleman R,Huber W. arrayQualityMetrics--a bioconductor package for quality assessment of microarray data*.* *Bioinformatics* 2009; **25**(3): 415-416.
3. 3. Ritchie M E, Phipson B, Wu D*, et al.* limma powers differential expression analyses for RNA-sequencing and microarray studies*.* *Nucleic Acids Res* 2015; **43**(7): e47.
4. 4. Robinson M D, McCarthy D J,Smyth G K. edgeR: a Bioconductor package for differential expression analysis of digital gene expression data*.* *Bioinformatics* 2010; **26**(1): 139-140.
5. 5. Leek J T, Johnson W E, Parker H S*, et al.* The sva package for removing batch effects and other unwanted variation in high-throughput experiments*.* *Bioinformatics* 2012; **28**(6): 882-883.
