# Supplementary material for: The Involvement of Intestinal Tryptophan Metabolism in Inflammatory Bowel Disease Identified by a Meta-Analysis of the Transcriptome and a Systematic Review of the Metabolome
Source: Nutrients. 2023 Jun 26;15(13):2886. doi: 10.3390/nu15132886 (PMC10346271; doi:10.3390/nu15132886)
Supplement: Supplementary file 1 [file nutrients-15-02886-s001.zip › supplementary data/Table S1.docx]

**Table S1** Characteristics of transcriptomics datasets selected for meta-analysis

|  | GEO ID/  ArrayExpress ID (publication year) | Sample size after removing outliers | Tissue location | Inflammation status of IBD patients | Platform | Adult/  Pediatric cohort |
| --- | --- | --- | --- | --- | --- | --- |
| 1 | GSE179285 (2021) | Terminal ileum from 62 CD and 8 controls;  Ascending/descending colon from 71 and 11 controls;  Sigmoid colon from 40 UC and 11 controls | Terminal ileum, ascending/descending colon, sigmoid colon | iCD: 33 active and 29 inactive  cCD: 11 active and 60 inactive  UC: 20 active and 20 inactive | Agilent-014850 Whole Human Genome Microarray 4x44K G4112F (GPL6480) | Adult |
| 2 | GSE137344 (2020) | 99 CD and 29 controls | ileum | 28 active and 71 inactive | NextSeq 550 (GPL21697) | Pediatric |
| 3 | GSE128682 (2020) | 28 UC, and 16 controls | Colon | 14 active and 14 inactive | NextSeq 550 (GPL21697) | Adult |
| 4 | GSE126124 (2019) | 38 CD, 18 UC, and 22 controls | colon | active | Affymetrix human gene 1.0 ST Array (GPL6244) | Pediatric |
| 5 | GSE102133 (2019) | 54 CD and 11 controls | Ileum | active | Affymetrix human gene 1.0 ST Array (GPL6244) | Adult |
| 6 | GSE112366 (2019) | 105 CD and 26 controls | Ileum | active | Affymetrix HT HG-U133+PM Array Plate (GPL13158) | Adult |
| 7 | GSE87466 (2018) | 86 UC and 20 controls | Colon | active | Affymetrix HT HG-U133+PM Array Plate (GPL13158) | Adult |
| 8 | E-MTAB-5790 (2018) | 36 CD and 32 controls | Terminal ileum | inactive | Agilent Whole Human Genome 4x44k Microarrays |  |
| 9 | GSE75214 (2017) | Colon from 97 UC, 8 CD patients, and 11 controls  Terminal ileum from 67 CD and 11 controls | Colon and terminal ileum | UC: 74 active and 23 inactive  cCD: 8 active  iCD: 51 active and 16 inactive | Affymetrix Human Gene 1.0 ST Array (GPL6244) | Adult |
| 10 | GSE83687 (2017) | 12 CD, 25 UC and 48 controls | colon | active | Illumina HiSeq 2500 (GPL16791) | Pediatric and adult |
| 11 | GSE68570 (2016) | 6 CD and 5 non-IBD controls | Ileal biopsies | active | Illumina HumanHT-12 V4.0 expression beadchip | Adult |
| 12 | GSE48958 (2015) | 13 UC and 8 controls | Colon | 7 active and 6 inactive | Affymetrix Human Gene 1.0 ST Array (GPL6244) | Adult |
| 13 | GSE57945 (2014) | 163 iCD, 55 cCD, and 42 controls | Ileum | active | Illumina HiSeq 2000 (GPL11154) | Pediatric |
| 14 | GSE47908 (2014) | 38 UC and 15 controls | Colon | active | Affymetrix Human Genome U133 Plus 2.0 Array (GPL570) | Adult |
| 15 | GSE52746 (2014) | 10 CD patients and 17 controls | Colon | active | Affymetrix Human Genome U133 Plus 2.0 Array (GPL570) | Adult |
| 16 | E-MTAB-184 (2012) | 24 CD, 61 UC and 19 controls | Colon | CD: 5 active and 19 inactive  UC: 21 active and 40 inactive | Illumina HumanHT-12 v3.0 Expression BeadChip | Adult |
