# Supplementary material for: The Involvement of Intestinal Tryptophan Metabolism in Inflammatory Bowel Disease Identified by a Meta-Analysis of the Transcriptome and a Systematic Review of the Metabolome
Source: Nutrients. 2023 Jun 26;15(13):2886. doi: 10.3390/nu15132886 (PMC10346271; doi:10.3390/nu15132886)
Supplement: Supplementary file 1 [file nutrients-15-02886-s001.zip › supplementary data/Table S3_meta-analysis.docx]

Table S3-1 Meta-analysis of 34 genes compared between active cUC and controls

| Gene | Pooled effect size (95% CI) | *p*-value | Tau^2^ | I^2^ | Test of heterogeneity  *p*-value |
| --- | --- | --- | --- | --- | --- |
| *SLC6A19* | -0.9741 (-1.6818, -0.2664) | 0.0131^*^ | 0.7292 | 86.5% | <0.0001 |
| *SLC16A10* | -0.0844 (-0.6072, 0.4384) | 0.7194 | 0.3369 | 73.8% | 0.0002 |
| *IDO1* | 2.2156 (1.1965, 3.2348) | 0.0010^*^ | 1.2899 | 87.8% | <0.0001 |
| *AFMID* | 0.4284 (-0.1673, 1.0241) | 0.1358 | 0.4445 | 78.1% | <0.0001 |
| *KYAT1* | 0.5544 (0.2797, 0.8291) | 0.0016^*^ | <0.0001 | 18.5% | 0.2782 |
| *KYAT2* | -0.5028 (-0.8463, -0.1593) | 0.0097^*^ | 0.0778 | 42.4% | 0.0844 |
| *KMO* | 1.3088 (0.7496, 1.8680) | 0.0006^*^ | 0.3001 | 73.2% | 0.0002 |
| *KYNU* | 2.5086 (1.2098, 3.8074) | 0.0021^*^ | 2.2441 | 89.9% | <0.0001 |
| *HAAO* | -0.3957 (-0.6747, -0.1168) | 0.0113^*^ | 0.0126 | 20% | 0.2649 |
| *ACMSD* | 0.0015 (-0.3175, 0.3205) | 0.9916 | 0.0834 | 42.9% | 0.0816 |
| *TPH1* | -0.7836 (-1.0780, -0.4891) | 0.0003^*^ | 0.0298 | 23.6% | 0.2339 |
| *AADC* | -1.9500 (-2.7035, -1.1966) | 0.0003^*^ | 0.7167 | 81.1% | <0.0001 |
| *AANAT* | 0.2418 (-0.4385, 0.9222) | 0.4284 | 0.5116 | 79.7% | <0.0001 |
| *SLC6A4* | -1.6970 (-2.3161, -1.0779) | 0.0002^*^ | 0.5185 | 81.0% | <0.0001 |
| *MAOA* | -2.0301 (-2.9794, -1.0807) | 0.0011^*^ | 0.9310 | 81.4% | <0.0001 |
| *MAOB* | -0.2952 (-0.5664, -0.0240) | 0.0364^*^ | 0.0269 | 17.4% | 0.2879 |
| *5-HTR_1B_* | 0.4247 (-0.2242, 1.0736) | 0.1656 | 0.4983 | 83.9% | <0.0001 |
| *5-HTR_1D_* | 0.2749 (-0.2332, 0.7831) | 0.2474 | 0.2930 | 71.7% | 0.0004 |
| *5-HTR_1E_* | -0.3406 (-0.6305, -0.0507) | 0.0274^*^ | 0.0068 | 7.5% | 0.3723 |
| *5-HTR_1F_* | -0.1652 (-0.6878, 0.3574) | 0.4791 | 0.3014 | 76.9% | <0.0001 |
| *5-HTR_2A_* | 0.3970 (0.0130, 0.7810) | 0.0443^*^ | 0.1473 | 58.4% | 0.0137 |
| *5-HTR_2B_* | 0.1522 (-0.2019, 0.5063) | 0.3505 | 0.0830 | 45.9% | 0.0636 |
| *5-HTR_3A_* | 0.4542 (-0.0453, 0.9538) | 0.0693 | 0.2997 | 73.6% | 0.0002 |
| *5-HTR_3B_* | -0.2451 (-0.6131, 0.1229) | 0.1631 | 0.1263 | 54.1% | 0.0259 |
| *5-HTR_3C_* | -1.4846 (-1.9126, -1.0565) | <0.0001^*^ | 0.2040 | 63.2% | 0.0054 |
| *5-HTR_3E_* | -1.1191 (-1.6080, -0.6303) | 0.0014^*^ | 0.1706 | 57.7% | 0.0277 |
| *5-HTR_4_* | -0.9267 (-1.3634, -0.4899) | 0.0012^*^ | 0.1968 | 61.6% | 0.0077 |
| *5-HTR_6_* | -0.3000 (-0.5150, -0.0851) | 0.0123^*^ | 0 | 0.0% | 0.6250 |
| *5-HTR_7_* | -0.2832 (-0.8627, 0.2963) | 0.2924 | 0.4484 | 79.9% | <0.0001 |
| *AhR* | 1.5596 (0.6458, 2.4734) | 0.0050^*^ | 0.9574 | 85.4% | <0.0001 |
| *ARNT* | -0.5642 (-0.9767, -0.1517) | 0.0135^*^ | 0.1806 | 63.5% | 0.0050 |
| *CYP1A1* | -0.0234 (-0.3074, 0.2606) | 0.8541 | 0.0351 | 24.5% | 0.2259 |
| *CYP1B1* | 0.4210 (0.0658, 0.7762) | 0.0257^*^ | 0.0845 | 45.0% | 0.0688 |
| *IL22* | 0.7847 (0.2259, 1.3435) | 0.0119^*^ | 0.3800 | 75.5% | <0.0001 |

* *P*<0.05, the pooled effect size is statistically significant.

Table S3-2 Meta-analysis of 34 genes compared between active cCD and controls

| Gene | Pooled effect size (95% CI) | *p*-value | Tau^2^ | I^2^ | Test of heterogeneity  *p*-value |
| --- | --- | --- | --- | --- | --- |
| *SLC6A19* | -0.0131 (-0.8865, 0.8604) | 0.9689 | 0.3127 | 67.5% | 0.0153 |
| *SLC16A10* | -0.4262 (-1.1649, 0.3125) | 0.1982 | 0.2443 | 58.2% | 0.0352 |
| *IDO1* | 2.6418 (1.0792, 4.2044) | 0.0074^*^ | 1.7093 | 84.2% | <0.0001 |
| *AFMID* | 0.4452 (-0.5606, 1.4510) | 0.3067 | 0.6052 | 72.1% | 0.0030 |
| *KYAT1* | 0.2607 (-0.7612, 1.2827) | 0.5409 | 0.7420 | 79.0% | 0.0002 |
| *KYAT2* | -0.9783 (-1.5574, -0.3991) | 0.0074^*^ | 0.1272 | 44.3% | 0.1097 |
| *KMO* | 0.8758 (0.4431, 1.3086) | 0.0035^*^ | 0.0118 | 6.8% | 0.3729 |
| *KYNU* | 2.5960 (0.9026, 4.2895) | 0.0110^*^ | 2.1401 | 87.1% | <0.0001 |
| *HAAO* | -0.2009 (-0.5438, 0.1420) | 0.1924 | 0.0034 | 0.0% | 0.5882 |
| *ACMSD* | -0.3278 (-0.6800, 0.0243) | 0.0621 | <0.0001 | 0.0% | 0.5641 |
| *TPH1* | -0.3704 (-1.0063, 0.2655) | 0.1946 | 0.1726 | 52.7% | 0.0607 |
| *AADC* | -1.4547 (-2.8993, -0.0102) | 0.0489^*^ | 1.5525 | 85.4% | <0.0001 |
| *AANAT* | 0.4699 (0.0873, 0.8525) | 0.0270^*^ | 0 | 0.0% | 0.5854 |
| *SLC6A4* | -1.5018 (-2.4335, -0.5702) | 0.0090^*^ | 0.5446 | 71.9% | 0.0032 |
| *MAOA* | -1.1518 (-2.4295, 0.1259) | 0.0683 | 1.1765 | 82.8% | <0.0001 |
| *MAOB* | -0.9661 (-1.7812, -0.1510) | 0.0285^*^ | 0.4127 | 69.6% | 0.0057 |
| *5-HTR_1B_* | 0.1597 (-0.9410, 1.2603) | 0.7077 | 0.6418 | 81.7% | 0.0002 |
| *5-HTR_1D_* | 0.0252 (-0.3257, 0.3760) | 0.8610 | 0 | 0.0% | 0.5625 |
| *5-HTR_1E_* | -0.1542 (-0.3911, 0.0828) | 0.1451 | 0 | 0.0% | 0.9172 |
| *5-HTR_1F_* | -0.1008 (-0.7123, 0.5107) | 0.6894 | 0.1748 | 52.7% | 0.0605 |
| *5-HTR_2A_* | 0.0777 (-0.1208, 0.2762) | 0.3607 | 0 | 0.0% | 0.9391 |
| *5-HTR_2B_* | 0.2035 (-0.4765, 0.8835) | 0.4765 | 0.2373 | 60.0% | 0.0287 |
| *5-HTR_3A_* | -0.2882 (-0.6942, 0.1177) | 0.1276 | <0.0001 | 3.5% | 0.3939 |
| *5-HTR_3B_* | -0.1051 (-0.8169, 0.6067) | 0.7198 | 0.2812 | 63.6% | 0.0174 |
| *5-HTR_3C_* | -1.3833 (-2.0801, -0.6864) | 0.0038^*^ | 0.2618 | 58.1% | 0.0358 |
| *5-HTR_3E_* | -1.0714 (-1.8129, -0.3298) | 0.0160^*^ | 0.2188 | 58.9% | 0.0453 |
| *5-HTR_4_* | 0.0848 (-0.6017, 0.7713) | 0.7636 | 0.2313 | 58.5% | 0.0341 |
| *5-HTR_6_* | -0.1058 (-0.6644, 0.4528) | 0.6469 | 0.1211 | 43.9% | 0.1128 |
| *5-HTR_7_* | -0.2380 (-0.8115, 0.3354) | 0.3347 | 0.0963 | 42.8% | 0.1198 |
| *AhR* | 1.4546 (0.4009, 2.5082) | 0.0164^*^ | 0.7935 | 79.9% | 0.0001 |
| *ARNT* | -0.1968 (-0.6683, 0.2747) | 0.3323 | 0.0484 | 24.7% | 0.2491 |
| *CYP1A1* | -0.0648 (-0.4560, 0.3264) | 0.6880 | 0.0462 | 5.6% | 0.3811 |
| *CYP1B1* | 0.8485 (-0.0531, 1.7502) | 0.0602 | 0.5405 | 74.6% | 0.0014 |
| *IL22* | 0.6831 (0.1047, 1.2616) | 0.0289^*^ | 0.1362 | 45.8% | 0.1006 |

* *P*<0.05, the pooled effect size is statistically significant.

Table S3-3 Meta-analysis of 32 genes compared between active iCD and controls

| Gene | Pooled effect size (95% CI) | *p*-value | Tau^2^ | I^2^ | Test of heterogeneity  *p*-value |
| --- | --- | --- | --- | --- | --- |
| *SLC6A19* | -0.8670 (-1.1472, -0.5868) | 0.0003^*^ | 0.0224 | 10.0% | 0.3526 |
| *SLC16A10* | -0.4768 (-0.5848, -0.3687) | <0.0001^*^ | 0 | 0.0% | 0.9816 |
| *IDO1* | 1.4627 (0.9819, 1.9434) | 0.0003^*^ | 0.1731 | 60.8% | 0.0181 |
| *AFMID* | 0.4117 (-0.0087, 0.8320) | 0.0536 | 0.1369 | 61.8% | 0.0153 |
| *KYAT1* | -0.6844 (-1.1351, -0.2337) | 0.0099^*^ | 0.1388 | 57.4% | 0.0287 |
| *KYAT2* | 0.6000 (0.4252, 0.7748) | 0.0002^*^ | 0 | 0.0% | 0.8281 |
| *KMO* | 0.1926 (-0.0428, 0.4281) | 0.0922 | <0.0001 | 0.0% | 0.5080 |
| *KYNU* | 0.9422 (0.6791, 1.2054) | 0.0001^*^ | 0.0124 | 0.0% | 0.4349 |
| *HAAO* | -0.8395 (-1.2625, -0.4165) | 0.0028^*^ | 0.1328 | 60.5% | 0.0189 |
| *ACMSD* | 0.2508 (-0.0738, 0.5753) | 0.1076 | 0.0382 | 30.3% | 0.1966 |
| *TPH1* | -0.2341 (-0.4392, -0.0290) | 0.0315^*^ | <0.0001 | 0.0% | 0.6754 |
| *AADC* | -0.8401 (-1.1659, -0.5143) | 0.0007^*^ | <0.0001 | 37.0% | 0.1460 |
| *AANAT* | 0.0274 (-0.3083, 0.3632) | 0.8420 | 0.0372 | 31.7% | 0.1978 |
| *SLC6A4* | -1.0224 (-1.4126, -0.6321) | 0.0007^*^ | 0.0470 | 44.2% | 0.0964 |
| *MAOA* | -1.0950 (-1.5881, -0.6020) | 0.0016^*^ | 0.1152 | 56.5% | 0.0320 |
| *MAOB* | -1.1350 (-1.6314, -0.6383) | 0.0014^*^ | 0.1767 | 67.3% | 0.0054 |
| *5-HTR_1D_* | -0.9948 (-1.3962, -0.5933) | 0.0009^*^ | 0.0934 | 53.2% | 0.0459* |
| *5-HTR_1E_* | -0.1129 (-0.4681, 0.2423) | 0.4512 | <0.0001 | 35.1% | 0.1734 |
| *5-HTR_1F_* | -0.1984 (-0.6050, 0.2083) | 0.2653 | 0.1093 | 66.5% | 0.0107 |
| *5-HTR_2A_* | -0.1654 (-0.4490, 0.1181) | 0.2032 | 0.0311 | 18.3% | 0.2905 |
| *5-HTR_2B_* | 0.5594 (0.2568, 0.8620) | 0.0040^*^ | 0.0294 | 21.8% | 0.2629 |
| *5-HTR_3A_* | -0.6870 (-0.9567, -0.4173) | 0.0008^*^ | 0.0096 | 4.8% | 0.3903 |
| *5-HTR_3B_* | -0.3967 (-0.7948, 0.0014) | 0.0505 | 0.0640 | 47.1% | 0.0924 |
| *5-HTR_3C_* | -0.9600 (-1.2148, -0.7051) | <0.0001^*^ | 0.0153 | 0.0% | 0.4590 |
| *5-HTR_3E_* | -0.7348 (-1.1320, -0.3376) | 0.0051^*^ | 0.0808 | 43.3% | 0.1167 |
| *5-HTR_4_* | -0.0694(-0.3932, 0.2544) | 0.6187 | <0.0001 | 39.6% | 0.1273 |
| *5-HTR_7_* | -0.1005 (-0.2628, 0.0618) | 0.1722 | 0 | 0.0% | 0.8695 |
| *AhR* | 1.0289 (0.3621, 1.6956) | 0.0092^*^ | 0.3933 | 78.4% | 0.0001 |
| *ARNT* | -0.2687 (-0.8127, 0.2754) | 0.2724 | 0.1794 | 62.1% | 0.0148 |
| *CYP1A1* | 0.0448 (-0.4027, 0.4923) | 0.8146 | 0.1551 | 70.7% | 0.0023 |
| *CYP1B1* | 0.7002 (0.5362, 0.8642) | <0.0001^*^ | 0 | 0.0% | 0.8719 |
| *IL22* | 0.6709 (0.5051, 0.8367) | <0.0001^*^ | 0 | 0.0% | 0.8646 |

* *P*<0.05, the pooled effect size is statistically significant.
