# Supplementary material for: The Involvement of Intestinal Tryptophan Metabolism in Inflammatory Bowel Disease Identified by a Meta-Analysis of the Transcriptome and a Systematic Review of the Metabolome
Source: Nutrients. 2023 Jun 26;15(13):2886. doi: 10.3390/nu15132886 (PMC10346271; doi:10.3390/nu15132886)
Supplement: Supplementary file 1 [file nutrients-15-02886-s001.zip › supplementary data/Table S4_metabolites summary.docx]

Table S4-1. Comparison of tryptophan levels in bio-samples derived from IBD patients and controls

| Study | Bio-sample | Results |
| --- | --- | --- |
| F. Di’Narzo *et al*. [2022] | **Serum** | No difference between groups |
| Notararigo *et al*. [2021] | **Serum** | UC < control |
| Gu *et al.* [2021] | **Serum** | Active UC < control |
| Scoville et al. [2018] | **Serum** | No difference between groups |
| Kolho *et al*. [2017] | **Serum** | Active UC < Active CD < control |
| Nikolaus *et al.* [2017] | **Serum** | Active CD < Inactive CD < control;  Active UC < control |
| Abautret-Daly *et al*. [2017] | **Plasma** | IBD < control |
| Kohashi *et al*. [2014] | **Serum** | Active UC < Inactive UC < control |
| Hisamatsu *et al*. [2012] | **Plasma** | Active IBD < Inactive IBD, CD < control |
| Gupta *et al*. [2012] | **Serum** | Active CD < Inactive CD < control |
| Ooi et al. [2011] | **Serum** | UC < CD < HC |
|  |  |  |
| F. Di’Narzo *et al*. [2022] | **Stool** | IBD > control, no difference between CD and UC |
| Wang *et al.* [2021] | **Stool** | Active CD showed increased level, but not significant |
| Franzosa *et al*. [2019] | **Stool** | CD > control, no difference between UC and control |
| Lloyd-Price *et al*. [2019] | **Stool** | No difference between groups |
| Bosch *et al*. [2018] | **Stool** | Active IBD > control, no difference between UC and CD |
| Kolho *et al*. [2017] | **Stool** | Active UC > Active CD > control |
| Lamas *et al*. [2016] | **Stool** | Inactive IBD < control |
|  |  |  |
| Diab *et al*. [2019] | **Colonic biopsy** | Active UC < Inactive UC = control |

Table S4-2 Metabolites produced through kynurenine pathway

| Study | Bio-sample | Results |
| --- | --- | --- |
| **Kynurenine (KYN)** |  |  |
| F. Di’Narzo *et al*. [2022] | **serum** | No difference between groups |
| Gu *et al.* [2021] | **serum** | KYN: Active UC < control  KYN/TRP: Active UC > control |
| Whiley *et al*. [2019] | **plasma** | UC > control |
| Abautret-Daly *et al*. [2017] | **plasma** | KYN: No difference  KYN/TRP: IBD > control, CD > control, Active IBD > control |
| Scoville et al. [2018] | **serum** | No difference between groups |
| Nikolaus *et al.* [2017] | **serum** | KYN: No difference between active CD, inactive CD and control, Active UC = Inactive UC > control;  KYN/TRP: Active CD > Inactive CD > control, Active UC = Inactive UC > control |
| Kohashi *et al*. [2014] | **serum** | Active UC > Inactive UC > control |
| Gupta *et al*. [2012] | **serum** | KYN: No difference  KYN/TRP: Active CD > Inactive CD = control |
|  |  |  |
| F. Di’Narzo *et al*. [2022] | **stool** | IBD > control, no difference between CD and UC |
| Kolho *et al*. [2017] | **stool** | Active UC > active CD > control |
| Lamas et al. [2016] | **stool** | Inactive IBD > control |
|  |  |  |
| Huhn *et al*. [2020] | **intestinal biopsy** | iCD < control, iCD < cCD, no difference between cCD and control, or between UC and control |
| Diab *et al*. [2019] | **colonic biopsy** | Active UC > Inactive UC = control |
|  |  |  |
| **Kynurenic acid (KA)** |  |  |
| F. Di’Narzo *et al*. [2022] | **serum** | CD < UC = control |
| Scoville et al. [2018] | **serum** | CD < non-IBD control |
| Kolho *et al*. [2017] | **serum** | Active CD < active UC < control |
| Nikolaus *et al.* [2017] | **serum** | Active CD = inactive CD < control  No difference between UC and control |
|  |  |  |
| F. Di’Narzo *et al*. [2022] | **stool** | No difference between groups |
| Franzosa *et al*. [2019] | **stool** | No difference between groups |
| Lloyd-Price *et al*. [2019] | **stool** | No difference between groups |
| Kolho *et al*. [2017] | **stool** | Active UC < Active CD |
|  |  |  |
| **Anthranilic acid (AA)** |  |  |
| F. Di’Narzo *et al*. [2022] | **serum** | No difference between groups |
| Nikolaus *et al.* [2017] | **serum** | Active CD > control  Active UC > Inactive UC |
|  |  |  |
| F. Di’Narzo *et al*. [2022] | **stool** | IBD > control, no difference between CD and UC |
|  |  |  |
| **Xanthurenic acid (XA)** |  |  |
| F. Di’Narzo *et al*. [2022] | **serum** | CD < UC = control |
| Whiley *et al*. [2019] | **plasma** | UC < control |
| Scoville et al. [2018] | **serum** | No difference between groups |
| Nikolaus *et al.* [2017] | **serum** | Active CD = Inactive CD < control  No difference between active UC, inactive UC and control |
|  |  |  |
| F. Di’Narzo *et al*. [2022] | **stool** | No difference between groups |
| Lloyd-Price *et al*. [2019] | **stool** | No difference between groups |
|  |  |  |
| **3-hydroxyanthranilic acid (3-HAA)** |  |  |
| Huhn *et al*. [2020] | **intestinal biopsy** | iCD > control, iCD > cCD, no difference between cCD and control, or between UC and control |
|  |  |  |
| **Picolinic acid (PA)** |  |  |
| F. Di’Narzo *et al*. [2022] | **serum** | IBD < control, no significant difference between CD and UC |
| Gu *et al.* [2021] | **serum** | No difference between UC and control |
| Whiley *et al*. [2019] | **plasma** | UC < control |
| Nikolaus *et al.* [2017] | **serum** | Active CD = Inactive CD < control  No difference between active UC, inactive UC and control |
| Yau *et al*. [2014] | **plasma** | No difference between groups |
|  |  |  |
| F. Di’Narzo *et al*. [2022] | **stool** | No difference between groups |
|  |  |  |
| Diab *et al*. [2019] | **colonic biopsy** | No difference between groups |
|  |  |  |
| **Quinolinic acid (QA)** |  |  |
| F. Di’Narzo *et al*. [2022] | **serum** | CD > UC, no difference between IBD and control |
| Gu *et al.* [2021] | **serum** | Active UC > control |
| Nikolaus *et al.* [2017] | **serum** | CD > UC > control  Active CD = Inactive CD > control  Active UC > control |
| Yau *et al*. [2014] | **plasma** | CD > UC = control |
|  |  |  |
| F. Di’Narzo *et al*. [2022] | **stool** | IBD > control, no difference between CD and UC |
|  |  |  |
| Diab *et al*. [2019] | **colonic biopsy** | Active UC > control, no difference between active UC and inactive UC, or between inactive UC and control |

Table S4-3 Metabolites produced through serotonin pathway

| Study | Biosample | Results |
| --- | --- | --- |
| **5-hydroxy-tryptophan (5-HTP)** |  |  |
| Gu *et al.* [2021] | **serum** | Discovery cohort: active UC < control  Validation cohort: no difference between UC and control |
| Lai *et al.* [2019] | **serum** | CD < HC, no difference between active and inactive CD |
|  |  |  |
| Lloyd-Price *et al*. [2019] | **stool** | No difference between groups |
| Kolho *et al*. [2017] | **stool** | Active UC > control, Active UC > active CD |
|  |  |  |
| **Serotonin (5-HT)** |  |  |
| F. Di’Narzo *et al*. [2022] | **serum** | No difference between groups |
| Gu *et al.* [2021] | **serum** | Active UC > control |
| Manzella *et al*. [2020] | **serum** | Active CD > inactive CD  No difference between active and inactive UC |
| Shajib *et al*. [2019] | **plasma** | active CD > inactive CD = control |
| Scoville et al. [2018] | **serum** | No difference between groups |
| Yu *et al*. [2016] | **plasma** | Inactive UC > control |
|  |  |  |
| F. Di’Narzo *et al*. [2022] | **stool** | No difference between groups |
| Lloyd-Price *et al*. [2019] | **stool** | No difference between groups |
|  |  |  |
| **5-hydroxyindoleacetic acid (5-HIAA)** |  |  |
| F. Di’Narzo *et al*. [2022] | **serum** | No difference between groups |
| Gu *et al.* [2021] | **serum** | Discovery cohort: active UC < control  Validation cohort: no difference between UC and control |
| Gu *et al.* [2021] | **serum** | No difference between groups |
| Scoville et al. [2018] | **serum** | No difference between groups |
| Yu *et al*. [2016] | **plasma** | Inactive UC > control |
|  |  |  |
| F. Di’Narzo *et al*. [2022] | **stool** | No difference between groups |
|  |  |  |
| Diab *et al*. [2019] | **colonic biopsy** | Active UC > Inactive UC = control |

Table S4-4 Metabolites produced through indole pathway

| Study | Biosample | Results |
| --- | --- | --- |
| **Indole** |  |  |
| F. Di’Narzo *et al*. [2022] | **stool** | CD > control, no difference between CD and UC, or between UC and control |
| De Preter *et al*. [2015] | **stool** | No difference between groups |
| Walton *et al*. [2013] | **stool** | CD > UC = control |
|  |  |  |
| **Tryptamine** |  |  |
| F. Di’Narzo *et al*. [2022] | **stool** | CD > control, no difference between CD and UC, or between UC and control |
| Gu *et al.* [2021] | **serum** | Active UC < control |
|  |  |  |
| **Indole acetic acid (IAA)** |  |  |
| F. Di’Narzo *et al*. [2022] | **serum** | serum: UC < control, no difference between CD and UC, or between CD and control |
| Gu *et al.* [2021] | **serum** | Active UC < control |
| Scoville et al. [2018] | **serum** | CD > UC = control |
|  |  |  |
| F. Di’Narzo *et al*. [2022] | **stool** | No difference between groups |
| Lloyd-Price *et al*. [2019] | **stool** | No difference between groups |
| Lamas et al. [2016] | **stool** | inactive IBD < control |
|  |  |  |
| **3-Methylindole (I3M)** |  |  |
| Lai *et al.* [2019] | **serum** | Inactive CD > control |
|  |  |  |
| F. Di’Narzo *et al*. [2022] | **stool** | UC < control, no difference between CD and UC, or between CD and control |
| De Preter *et al*. [2015] | **stool** | IBD < control |
|  |  |  |
| **Indole acrylic acid (IAcrA)** |  |  |
| Gu *et al.* [2021] | **serum** | Active UC < control |
| Lai *et al.* [2019] | **serum** | Active CD < control |
|  |  |  |
| **Indole propionic acid (IPA)** |  |  |
| F. Di’Narzo *et al*. [2022] | **serum** | No difference between groups |
| Gu *et al.* [2021] | **serum** | Active UC < control |
| Lai *et al.* [2019] | **serum** | Active CD < control |
| Alexeev et al. [2018] | **serum** | Active UC < control, no difference between active and inactive UC, or between inactive UC and control |
| Scoville et al. [2018] | **serum** | CD > UC, no difference between CD and HC or between UC and control |
|  |  |  |
| F. Di’Narzo *et al*. [2022] | **stool** | IBD showed decreased level, but not significant |
| Lloyd-Price *et al*. [2019] | **stool** | UC < control, CD showed decreased level, but not significant |
|  |  |  |
| **Indole acetaldehyde (IAAld)** |  |  |
| Gu *et al.* [2021] | **serum** | Active UC < control |
